# Exploring the predictors of academic performance: the role of personality, rational beliefs, and self-efficacy

**DOI:** 10.3389/fpsyg.2025.1650271

**Published:** 2025-08-01

**Authors:** Lucica Emilia Coşa, Vasile Cernat

**Affiliations:** Department of Teacher Training, “George Emil Palade” University of Medicine, Pharmacy, Sciences and Technology of Târgu Mureş, Târgu Mureş, Romania

**Keywords:** academic performance, self-efficacy, rational beliefs, aggressiveness, impulsive sensation-seeking

## Abstract

**Introduction:**

This study examines the predictive roles of personality traits, rational/irrational beliefs, and self-efficacy in academic performance, while also investigating how these factors interact with gender, residence, and school type.

**Methods:**

Data were collected from 453 students at George Emil Palade University of Medicine, Pharmacy, Science, and Technology in Târgu Mureş using the Zuckerman-Kuhlman Personality Questionnaire (ZKPQ), the General Self-Efficacy Scale (SES), and the short-form Attitudes and Beliefs Scale (ABSs).

**Results:**

Results revealed that institutional factors, particularly high school type, emerged as the strongest predictors of academic performance. Among the psychological traits, aggressiveness/hostility, impulsive sensation seeking, and rationality significantly predicted academic performance. Notably, impulsive sensation seeking was positively linked to higher performance in female but not male students, while aggressiveness/hostility predicted better performance only among students with high self-efficacy.

**Discussion:**

These findings highlight the potential for tailored intervention programs that take into account gender and personality differences to improve academic outcomes.

## 1 Introduction

Academic performance is a cornerstone of future professional success and overall life achievement (Kuncel et al., 2004). Given its significance, researchers have sought to identify key factors that influence academic outcomes (Mammadov, 2022; Abd-Elmotaleb and Saha, 2013). While some studies emphasize the role of personality traits (Mammadov, 2022), others highlight the contributions of self-efficacy and cognitive evaluations (Byars-Winston et al., 2017; Abd-Elmotaleb and Saha, 2013). Despite these insights, findings remain inconsistent, warranting further investigation into the psychological determinants of academic performance, particularly in exam settings. The present study addresses this gap by examining the alternative five-factor model of personality and the moderating effects of self-efficacy and rational/irrational beliefs.

The relationship between personality traits and educational success has been widely examined, particularly within the framework of the Big Five model (Poropat, 2009). Among these traits, conscientiousness consistently emerges as one of the strongest predictors of academic achievement (Rani Bhattacharjee and Ramkumar, 2015; Conard, 2006; Furnham et al., 2013; Hübner et al., 2022; O’Connor and Paunonen, 2007; Poropat, 2009; Verbree et al., 2023). Openness to experience also plays a significant role, likely due to its association with deep learning strategies (Chamorro-Premuzic and Furnham, 2008; Farsides and Woodfield, 2003).

Agreeableness has been linked to academic success, though its influence appears weaker (Poropat, 2009; Furnham et al., 2013; Farsides and Woodfield, 2003). Agreeable individuals tend to engage in collaborative learning, seek academic support, and provide emotional encouragement during assessments (Furnham et al., 2013).

Findings regarding neuroticism and extraversion are less consistent. While some studies report no significant association between neuroticism and academic performance (Mammadov, 2022), others indicate a negative correlation, particularly in primary education, but not at higher academic levels (Poropat, 2009). Moderating effects have also been observed; for instance, neuroticism may indirectly enhance performance at high self-efficacy levels while exerting a direct negative effect when self-efficacy is low (De Feyter et al., 2012).

How can these findings be explained? Some researchers propose that neurotic students experience heightened anxiety and diminished self-confidence, hindering their full engagement in learning (Chamorro-Premuzic, 2007). Others suggest that neuroticism may impair academic performance through ineffective learning strategies, specifically by hindering the ability to organize new information into coherent cognitive structures (Kokkinos et al., 2015).

The link between extraversion and academic performance appears most pronounced in primary education, where teacher-student relationships play a more central role in shaping outcomes (Poropat, 2011). At higher educational levels, assessment methods (e.g., written exams) and shifting motivations may weaken this association. Extraverted students may view studying instrumentally, as a means to obtain a degree or secure employment, rather than being intrinsically motivated (Komarraju et al., 2009). Additionally, their stronger focus on social engagement (O’Connor and Paunonen, 2007) could further detract from academic achievement, potentially leading to negative correlations in some contexts.

While most research on personality and academic performance has employed the Big Five model, few studies have utilized the alternative five-factor model (AFFM) (Stelmack, 2004). Evidence suggests significant correlations between factors across these two frameworks: sociability aligns with extraversion, neuroticism-anxiety with emotional stability, aggressiveness-hostility with openness and agreeableness, and activity with both extraversion and conscientiousness (Joireman, 2004; Zuckerman et al., 1993; Zuckerman, 2008).

Studies applying the AFFM reveal notable associations between aggressiveness and academic performance (Aluja-Fabregat and Torrubia-Beltri, 1998; Balkin, 1987; Lounsbury et al., 2003; Loveland et al., 2007). Research incorporating cognitive and temperamental factors further identifies sensation seeking, impulsivity, and fearlessness as key predictors (Colom et al., 2007). Gender differences also emerge, with evidence suggesting girls’ superior impulse control may contribute to stronger academic outcomes (Carvalho, 2016).

The link between impulsive sensation-seeking and academic performance is nuanced. Although traits like organization and routine tolerance typically facilitate learning, qualities seemingly at odds with impulsive sensation-seeking (Azikiwe, 1998; Cladellas et al., 2017; Cohen, 1991), exploratory learning methods may engage such individuals, enabling high achievement (Poropat, 2014; Gomá-i-Freixanet, 2001; Wismeijer and Gomà-i-Freixanet, 2012).

Caution is warranted in generalizing these findings, as personality profiles vary across academic disciplines (Coşa and Cernat, 2024). Person-environment fit theory posits that students self-select into fields aligning with their traits (Kaufman et al., 2013; Vedel, 2016), a congruence that enhances success (Wen et al., 2021; Vedel, 2016). Additionally, the influence of personality may differ by assessment type, with traits more strongly predicting performance in coursework than in final exams (Furnham et al., 2013).

Social cognitive theory views personality as a dynamic system of intrapersonal factors that motivate and regulate behavior (Bandura, 1991). Within this framework, self-efficacy emerges as a malleable construct developed through experience, particularly in challenging situations requiring sustained effort (Bandura, 2001). This theory posits that individuals’ performance outcomes depend fundamentally on their beliefs about their capability to execute specific tasks (Bandura, 1993). While some researchers suggest self-efficacy plays a relatively modest role in performance prediction (Heggestad and Kanfer, 2005), most studies emphasize its critical importance (Richardson et al., 2012; Talsma et al., 2018).

Meta-analytic research reveals only minor gender differences in academic self-efficacy (Huang, 2013), while consistently supporting a significant association between self-efficacy and academic achievement. These studies further identify various cognitive and non-cognitive factors that mediate or moderate this relationship, including reciprocal effects between self-efficacy and performance (Honicke and Broadbent, 2016). Notably, self-efficacy appears particularly beneficial for neurotic students’ academic performance (De Feyter et al., 2012), though evidence also suggests personality traits may themselves be mediated by self-efficacy (Stajkovic et al., 2018; Judge et al., 2007).

Rational Emotive Behavior Theory (Ellis et al., 2010) posits that belief systems mediate emotional responses to events. Irrational beliefs—characterized by rigidity and illogical extremes—tend to hinder goal attainment, while rational beliefs—flexible, logical, and pragmatic—facilitate achievement (Dryden and Branch, 2008). In academic contexts, these belief systems may indirectly influence performance through study behaviors. Specifically, irrational beliefs correlate with procrastination, subsequently impairing performance through rushed preparation or test anxiety (Balkis, 2013). While some studies report no direct link between irrational beliefs and objective academic outcomes (Allen et al., 2017), the association between negative affect and impaired performance is well-established (Callaghan and Papageorgiou, 2014). Conversely, rational beliefs consistently predict stronger academic results (Balkis et al., 2013; Balkıs, 2015). Students endorsing rational academic beliefs typically begin exam preparation earlier, thereby avoiding procrastination and achieving better outcomes through more effective study periods (Balkis et al., 2024; Sapp, 1996).

This review highlights persistent gaps and contradictions in understanding how personality traits and related factors influence academic performance. Future research should employ more nuanced analyses examining not only individual traits but also their interactions. Particularly valuable would be investigations using the alternative five-factor model (AFFM), which offers a distinct perspective from the dominant Big Five framework, while also accounting for contextual variables like gender and educational background. Additionally, given Romania’s pronounced rural-urban educational disparities (Munteanu, 2024), students’ geographical residence represents a critical moderating variable that may shape personality-performance relationships. Addressing these dimensions could resolve existing theoretical conflicts and provide a more comprehensive model of academic achievement determinants.

Objectives and hypotheses

General objective

This study aims to investigate the predictive role of personality traits in academic performance on the high school graduation examination, and to examine the potential moderating effects of self-efficacy and rational/irrational beliefs on these relationships.

Specific objectives

I. Confirmatory objectives

1. To assess the direct effects of personality traits, self-efficacy, and rational/irrational beliefs on graduation exam performance.
2. To evaluate whether self-efficacy and rational/irrational beliefs moderate the relationship between personality traits and academic performance.

II. Exploratory objectives

1. To investigate gender differences (male vs. female students) in the associations between psychological variables (personality, self-efficacy, rational/irrational beliefs) and exam performance.
2. To analyze how residential environment (urban vs. rural) interacts with psychological variables to influence academic outcomes.

Research hypotheses

H1: Personality traits, self-efficacy, and rational/irrational beliefs significantly predict performance on the high school graduation examination.

H2: The relationship between personality traits and academic performance is moderated by self-efficacy and rational/irrational beliefs.

The exploratory objectives do not include *a priori* hypotheses but will be examined through data-driven analyses to identify potential patterns related to gender and residential environment.

## 2 Materials and methods

### 2.1 Participants

Throughout the duration of this study, ethical protocols were followed, and participation in the research was voluntary. All participants were required to provide written informed consent, and the confidentiality and anonymity of their data were guaranteed. We used a cross-sectional research design and collected data from 453 students; 157 were boys, 295 were girls, and one participant did not specify their gender. The participants are first-year students from the Department for Teacher Training at the “G.E. Palade” University of Medicine, Pharmacy, Science, and Technology of Târgu Mureş, who have just graduated from high school. In Romania, the Departments of Teacher Education prepare students who simultaneously pursue their primary academic majors. Consequently, the sample includes students studying diverse scientific disciplines, including medicine, engineering, economics, law, history, and sports.

### 2.2 Measures

The study utilized the Zuckerman–Kuhlman Personality Questionnaire (ZKPQ), which was adapted for the Romanian population by Opre and Albu (2010) and Sârbescu and Neguţ (2013). Zuckerman defined the five factors from the alternative factor model as follows (Zuckerman et al., 1993):

- Impulsive sensation seeking: Consists of two facets: impulsivity and sensation seeking. Impulsivity refers to a lack of planning and a tendency to act quickly on impulse without consideration. Sensation-seeking describes a general desire for thrills or the willingness to take risks for excitement, a preference for unpredictable friends and situations, and a need for change and novelty (Cronbach’s alpha = 0.760).
- Neuroticism-anxiety: Describes emotional upset, fearfulness, tension, worry, lack of self-confidence, sensitivity to criticism, and obsessive indecision (Cronbach’s alpha = 0.869).
- Aggression-hostility: Depicts readiness to express verbal aggression, rude antisocial behavior, vengefulness, spitefulness, a quick temper, and impatience with others (Cronbach’s alpha = 0.632).
- Sociability: Differentiated into two aspects: enjoying large social events, interacting with many people, having many friends, and intolerance for social isolation (Cronbach’s alpha = 0.784).
- Activity: Consists of two facets: the need for general activity, impatience, and restlessness when there is nothing to do, and a preference for challenging and hard work, and an active and busy life (Cronbach’s alpha = 0.718).

The General Self-Efficacy Scale (SES) by Schwarzer and Jerusalem (1995), adapted and standardized for the Romanian population (Vasiliu et al., 2015), allows for obtaining a global indicator of self-efficacy (Cronbach’s alpha = 0.921).

The Attitudes and Beliefs Scale short form (ABS-SV; David, 2007) consists of 8 items and measures rational and irrational evaluative beliefs as described by Ellis and Dryden (1997) (Cronbach’s alpha for irrationality = 0.764 and for rationality = 0.766).

Academic results were measured by the general average obtained in the baccalaureate exam. The exam takes place at the end of the 12th grade. The results obtained were categorized into four performance categories.

## 3 Results

The descriptive statistics and correlation coefficients are presented in Table 1. Impulsive sensation seeking and aggressivity were negatively associated with academic performance (*r* = −0.13, *p* < 0.01 and *r* = −0.17, *p* < 0.01, respectively). In contrast, rationality was positively associated with academic performance (*r* = 0.19, *p* < 0.01). Multiple regression analysis showed that these three variables explained 6.3% of the variance in academic performance. Impulsive sensation seeking had a marginal effect (β = −0.08, *p* = 0.11), whereas aggressivity and rationality had significant effects (β = −0.13, *p* < 0.01 and β = 0.17, *p* < 0.001, respectively).

**TABLE 1:** Means, standard deviations, and correlations.

Variable	M	SD	1	2	3	4	5	6	7	8
1. Academic performance	3.040	1.001								
2. Self-efficacy	3.382	6.526	0.067							
3. Impulsive sensation seeking	9.987	3.802	−0.125**	−0.021						
4. Neuroticism-anxiety	8.640	4.961	0.047	−0.257**	0.143**					
5. Aggressivity	5.499	2.958	−0.165**	−0.091	0.316**	0.354**				
6. Activity	9.075	3.304	0.006	0.165**	0.119*	−0.222**	−0.093*			
7. Sociability	6.932	3.746	−0.069	0.047	0.234**	−0.197**	0.067	0.216**		
8. Irrationality ABS	9.318	3.567	−0.047	−0.133**	0.066	0.320**	0.232**	−0.01	−0.055	
9. Rationality ABS	14.695	3.814	0.190**	0.110*	−0.083	−0.013	−0.096*	0.027	−0.035	0.255**

Mean ± SD are used to represent mean and standard deviation, respectively. * indicates *p* < 0.05. ** indicates *p* < 0.01.

Given the important role of student gender and residence highlighted in previous studies, the analysis also controlled for these variables. When gender and residence were included in the analysis, the total variance explained by the model increased to 8.1%. The rural versus urban residence of the students had a significant effect on academic performance (β = 0.12, *p* < 0.01), whereas the effect of gender was not significant (β = 0.07, *p* = 0.11).

The next step of the analysis was to test for the hypothesized interactions. The first tested interaction was between neuroticism and gender. As expected, the change in R^2^ was significant when the equation also included the interaction term [R^2^-change = 0.013, *F*(1, 443) = 5.809, *p* < 0.05]. While among female students, neuroticism had no significant effect on academic performance (*B* = −0.009, *p* = ns), among male students it had a significant positive effect (*B* = 0.041, *p* < 0.05), indicating that higher levels of neuroticism are associated with greater academic performance for this group.

**FIGURE 1:**
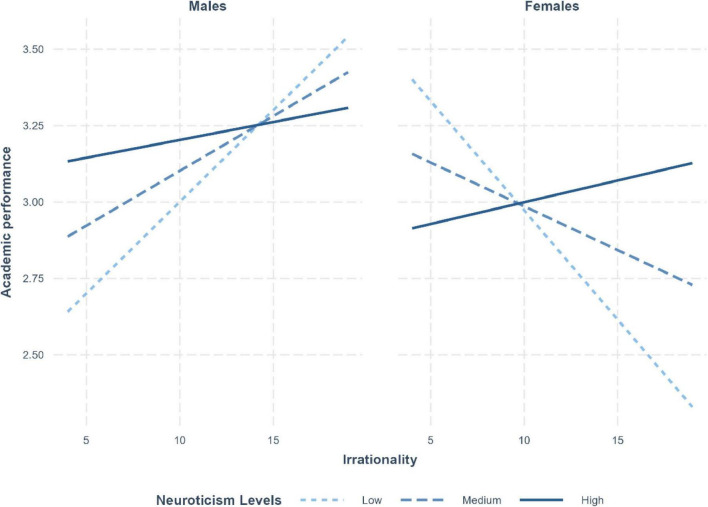
Three-way interaction effect of neuroticism (low, medium, high), irrational beliefs (*x*-axis), and gender (left: male; right: female) on academic performance (*y*-axis).

The interaction between neuroticism and gender on academic performance was qualified by a three-way interaction between these variables and participants’ irrationality [R^2^-change = 0.013, *F*(1, 433) = 7.41, *p* = 0.007]. As illustrated in Figure 1, participants’ neuroticism and irrationality interacted differently for the male and female students. Specifically, while the slopes of irrationality were significant for both male and female students only at low levels of neuroticism, the signs of these slopes were opposite: positive for male students and negative for female students (*B* = 0.06, *p* = 0.04 and *B* = −0.08, *p* = 0.01, respectively).

**FIGURE 2:**
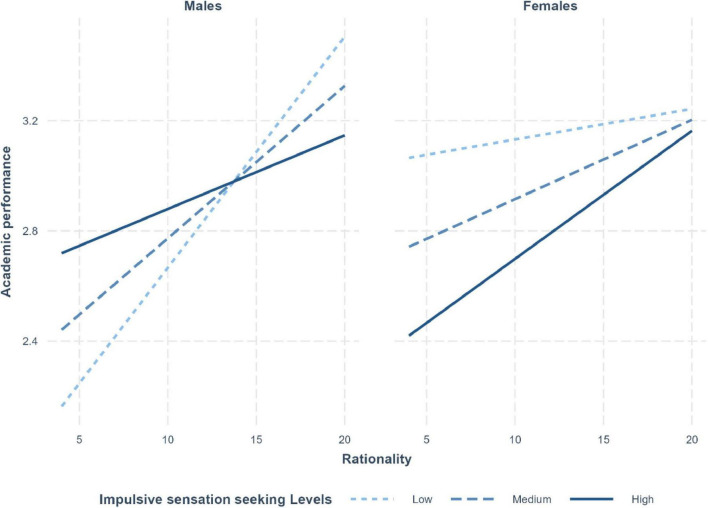
Three-way interaction effect of impulsive sensation seeking (low, medium, high), rational beliefs (*x*-axis), and gender (left panel: male; right panel: female) on academic performance (*y*-axis).

Gender also interacted marginally with impulsive sensation seeking and rationality [R^2^-change = 0.01, *F*(1, 433) = 4.17, *p* = 0.042]. As depicted in Figure 2, while all rationality slopes were positive, for men they were significant at medium and low levels of impulsive sensation seeking (*B* = 0.09, *p* = 0.01 and *B* = 0.06, *p* = 0.01, respectively), for females they were significant at medium and high levels of impulsive sensation (*B* = 0.03, *p* = 0.04 and *B* = 0.05, *p* = 0.01, respectively).

**FIGURE 3:**
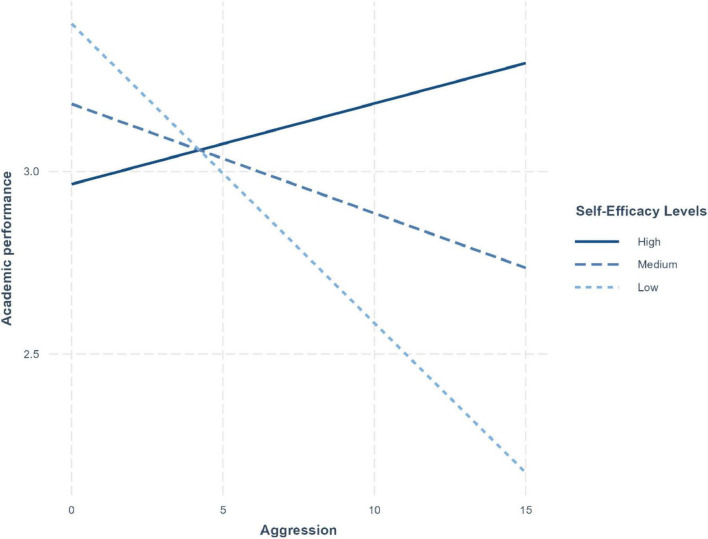
Two-way interaction between aggressivity (*x*-axis) and self-efficacy (low, medium, and high) on academic performance (*y*-axis).

Finally, the effects of aggressivity on academic performance depended on self-efficacy [R^2^-change = 0.02, *F*(1, 436) = 13.75, *p* < 0.001]. As illustrated in Figure 3, aggressiveness tended to have a positive effect on academic performance at higher levels of self-efficacy but negative effects at low levels of self-efficacy. However, only the last effect was significant (*B* = −0.08, *p* < 0.001). This two-way interaction was not qualified by students’ gender.

## 4 Discussion

This study examined two hypotheses concerning predictors of academic performance, operationalized through baccalaureate exam results in a sample of 453 Romanian students. Using three validated instruments, we assessed the Alternative Five Factor Model personality traits, self-efficacy, and rational/irrational beliefs. Our findings partially supported Hypothesis 1 (H1), revealing significant negative associations between academic performance and both impulsive sensation seeking and aggression/hostility. These results align with prior research (Balkin, 1987; Cladellas et al., 2017; Colom et al., 2007; Cohen, 1991; Lounsbury et al., 2003; Loveland et al., 2007; Poropat, 2014), though they contrast with Aluja-Fabregat and Torrubia-Beltri’s (1998) gender-specific findings regarding aggression.

However, when controlling for high school type, a proxy for prior academic achievement in Romania’s tracked education system (Munteanu, 2024), most personality effects became non-significant. Only rational beliefs maintained predictive power, suggesting their unique role in academic success, independent of institutional factors, gender, and residential environment.

The finding that personality traits accounted for only a modest proportion of variance in performance compared to high school type aligns with meta-analytic evidence underscoring the strong predictive role of prior academic achievement (Richardson et al., 2012). While the substantial influence of high school type highlights the importance of educational context over individual differences, it should be noted that high school type likely serves as a proxy not only for prior academic performance but also for cognitive and motivational factors that significantly shape academic performance.

Contrary to H1, our analysis found no significant associations between academic performance and the remaining personality factors (activity, neuroticism/anxiety, and sociability). These null findings align with Poropat’s (2009) and Mammadov’s (2022) conclusions for this age group, but contrast with studies reporting significant links between neuroticism (Chamorro-Premuzic, 2007; Kokkinos et al., 2015) or extraversion/sociability (Bidjerano and Dai, 2007; O’Connor and Paunonen, 2007; Komarraju et al., 2009) and academic achievement. Similarly, we found no direct relationship between self-efficacy and exam performance, a finding that contradicts some studies (Richardson et al., 2012; Talsma et al., 2018) but is consistent with others (Judge et al., 2007).

This discrepancy may reflect unique aspects of the Romanian educational context, where several factors could attenuate the predictive power of self-efficacy. First, the baccalaureate represents a high-stakes testing situation with significant consequences for university admission. This may create external pressures that override students’ self-efficacy beliefs. Second, this high-stakes context could further magnify the negative effects of test anxiety on performance (Richardson et al., 2012). These contextual factors may help explain why certain psychological predictors that emerge consistently in other educational systems failed to demonstrate significant effects in our study. Finally, we should also consider that this study employed a measure of general self-efficacy rather than one focused on academic performance.

Our second hypothesis (H2) proposed that rational/irrational beliefs and self-efficacy would moderate the relationship between personality traits and academic performance. The results partially support this hypothesis, revealing complex interaction patterns. Consistent with previous research (Balkis et al., 2024; Sapp, 1996), we found a general positive association between rational beliefs and academic performance. However, this relationship was moderated by both gender and impulsive sensation seeking. For male students, rational beliefs predicted better performance primarily at medium or low levels of impulsive sensation seeking. This aligns with Cohen’s (1991) assertion that academic success requires organization and planning–cognitive processes that rational, less impulsive individuals may implement more effectively. On the other hand, for female students rational beliefs enhanced performance even at medium-high levels of impulsive sensation seeking. This suggests that impulsive female students may compensate through exploratory learning strategies (Gomá-i-Freixanet, 2001; Poropat, 2014), while still benefiting from rational belief structures.

The analyses further showed that the aggression/hostility trait showed differential effects depending on self-efficacy levels. At high self-efficacy, aggression/hostility was positively associated with performance, suggesting these students may channel aggressive tendencies motivationally (Honicke and Broadbent, 2016). At low self-efficacy, aggression/hostility correlated with poorer performance, indicating maladaptive outcomes when combined with low confidence. These patterns were consistent across genders, though prior research suggests stronger effects in males (Aluja-Fabregat and Torrubia-Beltri, 1998).

In conclusion, our results support a dynamic conceptualization where traits manifest differently depending on cognitive/affective moderators, gender influences how personality-behavior relationships unfold, and high self-efficacy may help “reframe” typically negative traits (like aggression) into performance-enhancing factors.

### 4.1 Limitations

While this research offers valuable insights into personality-academic performance relationships, its findings are constrained by several limitations. The study’s reliance on self-report measures for assessing personality traits introduces potential response biases, including social desirability effects and subjective interpretations of questionnaire items, which may not fully capture the complexity of these psychological constructs. Furthermore, personality is a complex construct that cannot be fully captured through an evaluation tool such as the one used in the current study. Finally, the exclusive use of students from a single university limits the generalizability of our results to broader student populations. The non-representative nature of our sample suggests these findings should be complemented by future research employing more diverse, representative samples.

## 5 Conclusion

This study investigated the predictive relationships between personality traits (alternative five-factor model), rational beliefs, self-efficacy, and academic performance. Our findings revealed several key insights:

Initial analyses showed negative associations between academic performance and both impulsive sensation-seeking and aggression/hostility. However, after controlling for high school type (likely a proxy for prior academic achievement), gender, and residential environment, only rational beliefs maintained a significant direct relationship with performance. The strongest predictors emerged as institutional factors, particularly high school type, rather than individual psychological traits.

The study uncovered important interaction effects that qualify these relationships. The beneficial effect of rational beliefs on academic performance varied significantly by both gender and levels of impulsive sensation-seeking. Similarly, the impact of aggression/hostility depended crucially on students’ self-efficacy levels, demonstrating how motivational factors can modulate trait expression.

### 5.1 Implications

These results suggest three lines of action. First, the development of targeted personalized intervention programs that account for gender differences in how rational beliefs and personality traits influence learning outcomes. Second, training programs to enhance students’ academic self-efficacy, particularly for those displaying aggression/hostility traits, to help channel these characteristics productively. And third, greater support for students from rural areas, who demonstrated systematically lower performance despite comparable trait profiles.

## Data Availability

The raw data supporting the conclusions of this article will be made available by the authors, without undue reservation.
